# A Case of Lithium-Associated Hypocalciuric Hypercalcemia

**DOI:** 10.7759/cureus.10606

**Published:** 2020-09-23

**Authors:** Philip C Nwabufor, Oluwamayowa N Omoniyi, Samson O Oyibo

**Affiliations:** 1 General Medicine, Peterborough City Hospital, Peterborough, GBR; 2 Emergency Medicine, Peterborough City Hospital, Peterborough, GBR; 3 Diabetes and Endocrinology, Peterborough City Hospital, Peterborough, GBR

**Keywords:** lithium, hypocalciuric hypercalcemia, bipolar disorders, parathyroid gland, calcium, parathyroid hormone, urinary calcium, calcium creatinine clearance ratio

## Abstract

Lithium is the treatment of choice for acute manic, mixed, and depressive episodes of bipolar disorder, along with long-term prophylaxis. A significant proportion of patients taking lithium develop lithium-associated hypercalcemia. Most cases are due to lithium-associated hyperparathyroidism with underlying parathyroid adenoma or hyperplasia. We present a 67-year-old woman who presented with increasing lethargy and loss of concentration and was found to have slightly raised serum calcium levels with inappropriately low urinary calcium excretion levels characteristic of hypocalciuric hypercalcemia. She had been on lithium therapy for over 15 years for bipolar disease. She had no other cause for these findings and had no family history to suggest familial hypocalciuric hypercalcemia. Neck imaging ruled out any parathyroid adenoma or hyperplasia. A diagnosis of lithium-associated hypocalciuric hypercalcemia was discussed with the patient, and she remains stable under surveillance.

## Introduction

Lithium has been used for the treatment of bipolar disorder for more than 60 years, and current guidelines recommend lithium as the treatment of choice for acute manic, mixed, and depressive episodes of bipolar disorder, along with long-term prophylaxis [1,2]. The aim of treatment is to reduce the severity and number of episodes of depression and mania to allow as normal life as possible.

The prevalence of hypercalcemia in individuals who were on lithium therapy for bipolar disorder has been reported as being up to 25%-30% [3,4]. This included cases where a parathyroid adenoma or hyperplasia had been found and those cases without any parathyroid lesion. Lithium interacts with and renders the calcium-sensing receptors (CaSR) in the parathyroid glands and kidneys less sensitive to hypercalcemia, such that a higher threshold level of serum calcium is required to suppress parathyroid hormone release and to suppress renal tubular calcium reabsorption in the parathyroid glands and kidneys, respectively [3]. The increased renal reabsorption of calcium results in hypocalciuria and mild hypercalcemia, a condition we called lithium-associated hypocalciuric hypercalcemia (LAHH). Serum calcium should be checked before starting treatment, six months after and at least yearly in patients on lithium therapy [5,6]. Stopping lithium therapy usually improves the calcium levels, but this is not always possible during optimum control of the bipolar disorder because of the high risk of relapse [7].

There are very few case reports titled LAHH. This is likely due to underdiagnosis or underreporting. Additionally, the term “hypocalciuric hypercalcemia” is usually related to familial hypocalciuric hypercalcemia (FHH). We present a case of a female patient who developed LAHH while on long-term lithium therapy for bipolar disorder.

## Case presentation

Medical history and demographics

A 67-year-old Caucasian woman presented with a three-month history of feeling unwell with lethargy, increased thirst, and loss of concentration. Her serum calcium level had been slightly raised in the six months prior to this presentation. Her medical history included bipolar disorder for over 15 years, for which she took lithium carbonate 600 mg daily, hypothyroidism for which she took levothyroxine 100 mcg daily, idiopathic pulmonary hypertension for which she took bosentan 125 mg twice a day, and obstructive sleep apnea for which she used a continuous positive airway pressure apparatus. She had no family history of hypercalcemia. General examination did not reveal any abnormal findings. She had a body mass index of 29.5 kg/m^2^.

Investigations

Initial investigations revealed a slightly raised serum calcium level, slightly reduced serum phosphate, and a slightly raised parathyroid hormone level. The patient’s hemoglobin, thyroid hormone, and liver enzymes levels were normal. The finding of an inappropriately low 24-hour urinary calcium excretion and low calcium creatinine clearance ratio (CCCR) confirmed hypocalciuric hypercalcemia. A normal glycated hemoglobin level excluded diabetes mellitus (Table 1).

**Table 1:** Initial blood and urine investigations at presentation Demonstrating mild hypercalcemia, mild hypophosphatemia, a slightly raised parathyroid hormone level, and relative hypocalciuria.

Test	Normal values	Patient’s results
Sodium (mmol/L)	132-145	139
Potassium (mmol/L)	3.4-5.1	4.9
Creatinine (mmol/L)	45-84	97
Estimated glomerular filtration rate (ml/min)	>60	57
Hemoglobin (g/L)	115-165	145
Erythrocyte sedimentation rate (mm//hr)	0-29	5
Corrected calcium (mmol/L)	2.20-2.60	2.69
Phosphate (mmol/L)	0.8-1.5	0.72
Magnesium (mmol/L)	0.7-1.0	0.89
Glycated hemoglobin (mmol/mol)	<42	35
25-hydroxy vitamin D (nmol/L)	>50	50
Alkaline phosphatase (U/L)	30-130	83
Thyroid stimulating hormone (mU/L)	0.3-4.2	0.93
Parathyroid hormone (pmol/L)	1.4-6.2	7.4
Serum lithium concentration (mmol/L)	0.4-1.0	0.9
24-hour urine calcium (mmol/L)	2.5-7.5	1.3
Calcium creatinine clearance ratio	>0.01	0.0078

**Figure 1:**
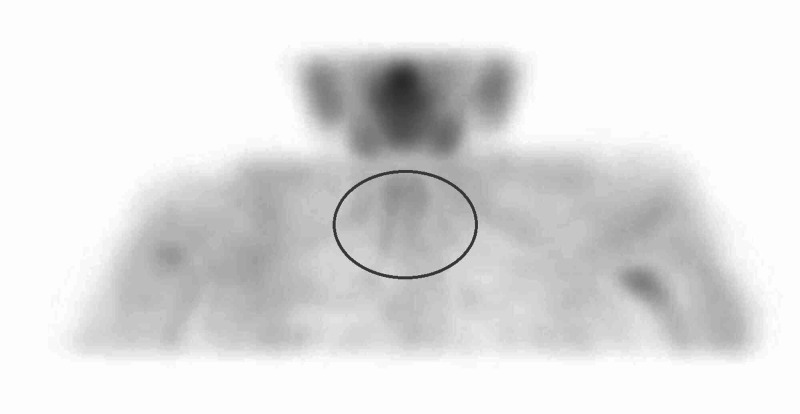
A sestamibi parathyroid scan showing no increased uptake within the region of the parathyroid glands (circle)

Serum Bence-Jones proteins and protein electrophoresis were normal. Chest x-ray revealed longstanding right-sided hilar prominence with some linear atelectasis. An ultrasound and nuclear medicine (sestamibi) scan of the neck did not reveal any features to suggest the presence of a parathyroid adenoma or hyperplasia (Figure 1). A renal ultrasound revealed normal kidney size and anatomy with no stones. Previous routine blood results were normal apart from mild hypercalcemia in the last six months.

Treatment

A diagnosis of LAHH was made. This diagnosis was discussed with the patient, and she was informed that there was no specific treatment, especially as her serum calcium level was only slightly raised.

Outcome and follow-up

The patient remains under surveillance with mildly raised serum calcium levels (2.63-2.67 mmol/L) and a CCCR less than 0.01. Her kidney function remains stable. However, the patient complained of ongoing tiredness, which could have been related to the chronic mild hypercalcemia.

## Discussion

We have described a patient with chronic mild hypercalcemia, slightly raised parathyroid hormone levels, and inappropriately low urinary calcium levels. She had no other cause for these biochemical findings. There was no evidence of a parathyroid adenoma or hyperplasia. She had been on lithium therapy for 15 years with normal serum calcium levels up until six months before this presentation. We believe that these features were suggestive of acquired hypocalciuric hypercalcemia secondary to lithium therapy.

There are several differential diagnoses to consider in our patient. FHH is a rare autosomal dominant condition where mutations in the *CaSR* gene cause decreased sensitivity of the CaSR in the kidneys and the parathyroid glands. Patients are generally below the age of 30 years and have a family history of hypercalcemia [8]. FHH is usually asymptomatic and we assumed the same for LAHH; however, further meta-analysis is required to assess this further. Upregulation of the CaSR within pulmonary arterial smooth muscle cells has been causally linked to pulmonary arterial hypertension through excess intracellular calcium-mediated smooth muscle cell proliferation [9,10]. However, data concerning reports of hypocalciuric hypercalcemia in patients with pulmonary arterial hypertension are rare. Bosentan is an endothelin receptor antagonist. A study in rats demonstrated that bosentan prevented the parathyroid cell proliferation normally associated with low calcium diet-induced hyperparathyroidism in rats [11]. However, data concerning reports of bosentan affecting serum calcium levels are rare. There has been a single case of reversible hypocalciuric hypercalcemia in a patient with untreated hypothyroidism. This resolved once thyroid replacement treatment was started [12]. Further reports of this phenomenon are rare, and our patient was receiving adequate thyroid hormone replacement. The link between obstructive sleep apnea and low vitamin D levels with resultant mild hyperparathyroidism has been investigated. However, our patient had a normal vitamin D level [13].

Parathyroid adenomas and multiglandular hyperplasia requiring parathyroidectomy have been found in patients with hypercalcemia secondary to lithium use [14,15]. It remains unclear whether these were cases of underlying primary hyperparathyroidism exacerbated by lithium use or whether the lithium-induced disruption of the CaSR-induced parathyroid tissue proliferation [16]. Cases of hypocalciuric hypercalcemia in patients on lithium therapy without any discernible parathyroid nodule or hyperplasia should be in a distinct group labeled LAHH, which is biochemically similar to FHH. A combination of parathyroid ultrasonography, nuclear medicine imaging, and urinary CCCR estimation is required to diagnose true LAHH, as cases of LAHH will not respond to parathyroidectomy. Genetic testing for FHH should still be performed in patients with suspected LAHH if they have a family history of hypercalcemia.

Our patient continued to complain of tiredness. We did not know if the symptoms were related to the mild hypercalcemia. Stopping the lithium therapy was not an option because of her well-controlled bipolar disorder, as discussed with her psychiatrist. The possible use of calcimimetic drugs, which have been successfully used in treating hypercalcemia associated with primary and secondary hyperparathyroidism and also FHH, needs to be explored [17]. 

## Conclusions

LAHH is a distinct entity with similar biochemical features to FHH. This underreported condition needs to be considered when there are features of mild hypercalcemia, slightly raised parathyroid hormone levels, and relative hypocalciuria without a parathyroid lesion in a patient who is on lithium therapy for bipolar disorder. All such cases should be referred to the endocrinologist for further evaluation.
